# Predicting the extinction of Ebola spreading in Liberia due to mitigation strategies

**DOI:** 10.1038/srep12172

**Published:** 2015-07-20

**Authors:** L. D. Valdez, H. H. Aragão Rêgo, H. E. Stanley, L. A. Braunstein

**Affiliations:** 1 Departamento de Física, Facultad de Ciencias Exactas y Naturales, Universidad Nacional de Mar del Plata by Instituto de Investigaciones Físicas de Mar del Plata (IFIMAR-CONICET), Deán Funes 3350, 7600 Mar del Plata, Argentina; 2 Departamento de Física, Instituto Federal de Educação, Ciência e Tecnologia do Maranhão, São Lu s, MA, 65030-005, Brazil; 3 Center for Polymer Studies, Boston University, Boston, Massachusetts 02215, USA

## Abstract

The Ebola virus is spreading throughout West Africa and is causing thousands of deaths. In order to quantify the effectiveness of different strategies for controlling the spread, we develop a mathematical model in which the propagation of the Ebola virus through Liberia is caused by travel between counties. For the initial months in which the Ebola virus spreads, we find that the arrival times of the disease into the counties predicted by our model are compatible with World Health Organization data, but we also find that reducing mobility is insufficient to contain the epidemic because it delays the arrival of Ebola virus in each county by only a few weeks. We study the effect of a strategy in which safe burials are increased and effective hospitalisation instituted under two scenarios: (i) one implemented in mid-July 2014 and (ii) one in mid-August—which was the actual time that strong interventions began in Liberia. We find that if scenario (i) had been pursued the lifetime of the epidemic would have been three months shorter and the total number of infected individuals 80% less than in scenario (ii). Our projection under scenario (ii) is that the spreading will stop by mid-spring 2015.

For a fleeting moment last spring, the epidemic sweeping West Africa might have been stopped. But the opportunity to control the virus, which has now caused more than 7,800 deaths, was lost^1^.

The current Ebola outbreak in Western Africa is one of the deadliest and most persistent of epidemics^2^. According to World Health Organization data^3^ as of 31 December 2014 there have been 20,171 cases and 7,889 deaths in three countries alone: Guinea, Sierra Leone, and Liberia. These numbers increase when cases and deaths from countries in which the outbreak has been officially declared over^4^ are included.

Cultural, economic, and political factors in that region of Western Africa^2^,^5^,^6^,^7^,^8^,^9^ have hampered the effectiveness of the intervention strategies used by the health authorities. Because of a lack of reliable information about local patterns of the spreading of the Ebola virus disease (EVD)^10^,^11^,^12^, the strategies currently being used, including the mobilisation of resources, the creation of new Ebola treatment centers (ETC), the development of safe burial procedures, and the international coordination of the efforts^13^ as of 1 January 2015 have been only partially successful.

Legrand *et al.*^14^, developed a seminal mathematical stochastic model with full mixing that reproduces the 1995 EVD outbreak in the Congo and the 2000 outbreak in Uganda. The population is divided into six compartments. Individuals in the susceptible compartment transition to exposed compartment and to the infectious compartment when they become infected. A percentage of these infected individuals are hospitalised and there are two possible outcomes: (i) they die, but before they are removed from the epidemic system they transition into the funeral compartment and infect other susceptible individuals, or (ii) they are removed from the system because they are cured. The maximum likelihood method is used to calibrate the model with the data.

Rivers *et al.*^15^, used a deterministic version of this model and least-squares optimisation to fit the current Liberia and Sierra Leone outbreak data. Their model indicated that the epidemic would not reach its peak until 31 December 2015. Gomes *et al.*^16^ estimated the transmission coefficients using the model provided by Legrand *et al.*^14^, a Global Epidemic and Mobility model that uses a structured metapopulation scheme, integrating the stochastic modelling of the disease dynamic, high resolution census and human mobility patterns at the global scale using a high resolution population data^17^,^18^. The parameters were estimated by fitting the total number of cumulative deaths from Liberia, Sierra Leone, and Guinea during the period 6 July–9 August 2014. The transmission parameters obtained were used to forecast three months of EVD propagation in West Africa and the probability of its spreading internationally. They found that the risk of cases spreading to other countries was low. Poletto *et al.*^19^ used the same model and found that reducing the number of travellers crossing international boundaries delays the arrival of EVD by only a few weeks. Merler *et al.*^20^ used methods similar to those in Ref. 16 to model the effect of epidemic spreading between geographical regions. They took into account the movements of non-infected individuals who were assisting in health-care facilities, those who took care of non-hospitalised infected individuals, and those who attended funerals.

Population mobility—the movement of individuals seeking safer areas, better health infrastructures, or food supplies—strongly affects disease propagation and plays a major role in epidemic spreading and in the effectiveness of any intervention scheme^21^. In Liberia, 54% of the population over the age of 14 are internally displaced^22^. Understanding these patterns of movement is essential when planning interventions to contain regional outbreaks. In recent years a number of mobility studies have been published^23^,^24^,^25^, including Wesolowski *et al.*^21^, who used mobile telephone network data to analyse mobility patterns that could be useful to understand the Ebola outbreak. They analysed data sources from mobile phone call records (CDRs), national census microdata samples, and spatial population data in order to estimate domestic and international mobility patterns in West African countries. The best mobility estimates were obtained for Senegal, Cote d’Ivoire, and Kenya, and Wesolowski *et al.*^21^,^25^ used them to produce a spatial interaction model of national mobility patterns in order to estimate how the EVD affected regions are connected by population flows.

We use a stochastic compartmental model and a set of differential equations, which are the quasi-deterministic representation of a stochastic model, to understand how population mobility affects the spreading of EVD between regions (counties) within Liberia. Our model quantifies how mobility between counties affects epidemic spreading inside Liberia, and we find that although reducing mobility among counties delays the spread of Ebola, it does not contain it. Our study indicates that the response implemented in August 2014 will result the extinction of the epidemic by mid-spring 2015, but it also indicates that an earlier response would have been extremely effective in containing the disease.

## Results

### Model

In our model we classify individuals as susceptible (S), exposed (E), i.e., infected but not infectious, infected (I), hospitalised (H), recovered (R), i.e., either cured or dead with a safe burial that does not transmit the disease, or dead (F) with an unsafe burial that transmits the disease. We also classify infected and hospitalised individuals according to their fate: those who are infected, will be hospitalised, and will die (I_DH_), those who are infected, won’t be hospitalised, and will die (I_DNH_), those who are infected, will be hospitalised, and will recover (I_RH_), those who are infected, won’t be hospitalised, and will recover (I_RNH_), those who are hospitalised and will die (H_D_), and those who are hospitalised and will recover (H_R_). The symbols S, E, I, H, R, and F indicate both the classification and the population percentage within the classification.

**Figure 1:**
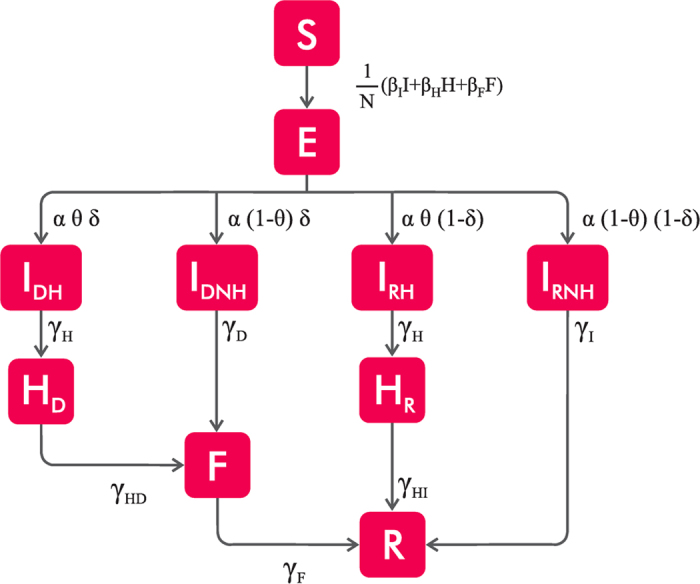
A schematic of the transitions between different states of our model for the EVD spreading in West Africa 2014 and their respective transition rates. In the model, the population is divided into ten compartmental states (See Table S1): Susceptible (*S*) individuals who in contact with infected individuals can become exposed (*E*). These *E* individuals after the incubation period become infected and follow four different scenarios: (i) Infected individuals that will be cured—recovered—without hospitalisation (I_RNH_); (ii) Infected individuals who will be cured (I_RH_) after spending a period on a hospital (*H*_*R*_); (iii) Infected individuals without being hospitalised (I_DNH_) who will die and may infect other individuals in their funerals (*F*); and (iv) Infected individuals (I_DH_) that even after spending a period in a hospital (*H*_*D*_) will die and may also spread the infection in the funerals (*F*). Recovered individual (*R*), can be cured or dead.

Figure 1 shows a schematic presentation of the model indicating the compartmental states (red boxes) and the transition rates among the states (connecting arrows). The *I*, *I* = *I*_*DH*_ + *I*_*DNH*_ + *I*_*RH*_ + *I*_*RNH*_ represents the total number of infected individuals, and *H* = *H*_*R*_ + *H*_*D*_ the total number of those hospitalised. Table 1 shows the different parameters used to calculate the transition rates among the different compartmental states, and Table S1 (see Supplementary Information) shows the *N*_*T*_ = 12 transitions between states and their rates λ_*i*_ with *i* = 1, … *N*_*T*_.

To determine how geographic mobility spreads the disease, we utilise the model of West African regional transportation patterns developed by Wesolowski *et al.*^21^,^25^. In their research they applied a gravity model to mobile phone data for Senegal to estimate the flow of individuals between counties in Liberia. Although these movement data are “historical” and do not reflect how local population behaviour may have changed in response to the current crisis, we assume the patterns of mobility obtained in the Wesolowski model^21^,^25^ still represent a good approximation of the routine commuting patterns of the population in Liberia prior to the outbreak. This is different to post-outbreak models that describe travel patterns that reflect human efforts to avoid the disease or to attend funerals of epidemic victims (see Ref. 20 and references in therein).

We assume that there is a flow of individuals between all *N*_co_ = 15 counties of Liberia, and that only susceptible or exposed individuals can travel between counties. Thus the deterministic evolution equations for the number of individuals in each state in county *c* in our model are
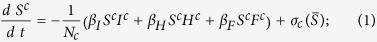

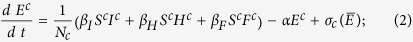

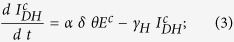

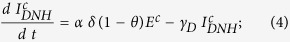

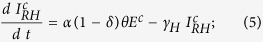

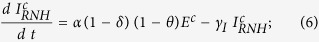

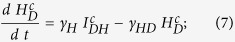

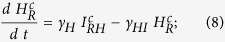

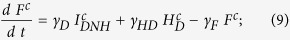

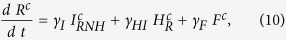
where *σ*_*c*_ is the total rate of mobility in each county *c* and is given by 
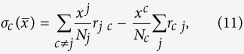
where *x*^*j*^ (*x*^*c*^) is the number of individuals (susceptible or exposed), in county *j* (*c*), *N*_*j*_ (*N*_*c*_) the total population of county *j* (*c*), and *r*_*jc*_ and *r*_*cj*_ the mobility rates from county *j* → *c* and from county *c* → *j*, respectively. Note that due to mobility the population in each county changes, but since this evolution is much slower than the dynamics of the disease spreading, we consider *N*_*c*_ to be constant (in our model without restriction on the mobility, the population in each county changes less than a 5% each year). In addition, in this model we disregard the mobility inside each county, i.e., we assume that the population is fully mixed. Because recovered individuals are unable to transmit the disease or be reinfected, they do not affect the results of our model and we disregard their movements between counties.

In the context of complex network research, this model of mobility between counties breaks the traditional full-mixing approach because each county can be thought of as a node of a metapopulation network^31^ in which the weight of each link is proportional to the mobility flow. Note that if in Eqs (1, 2, 3, 4, 5, 6, 7, 8, 9, 10) we drop the index *c* and disregard the flow mobility we are no longer taking the counties into account, and we have a scenario that represents the spread throughout the entire country.

### Transmission rates estimated

**Figure 2:**
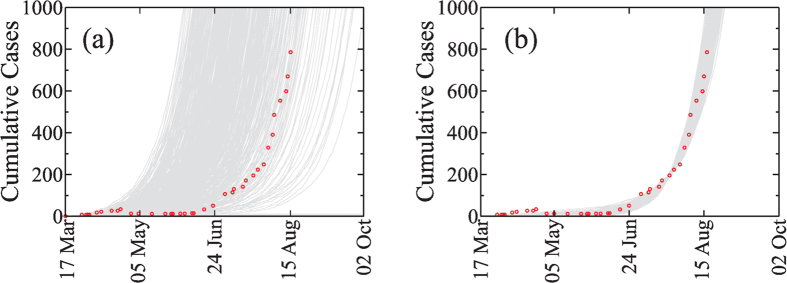
Cumulative number of cases in Liberia for the parameters given in **Table** 1. Cumulative number of cases obtained with our stochastic model with the transition presented in Table S1 and Eq. (11) in Liberia with 1000 realisations (gray lines) and the data (symbols) without temporal shift (**a**) and (**b**) with a temporal shift using *s*_*c*_ = 200. The transmission coefficients *β*_*I*_ = 0.14, *β*_*H*_ = 0.29 and *β*_*F*_ = 0.40 were obtained as explained in *Methods: Calibration with the deterministic equations*. From the WHO’s data the index case is located at Lofa on March 17 2014.

According to WHO data^3^, the first index case (patient zero) was diagnosed in Lofa on 17 March 2014. Thus our initial conditions in Lofa are (i) one infected individual in that county and (ii) the rest of the population susceptible. The estimated rates of transmission in day^−1^ obtained (using the method presented in the section *Methods: Calibration with the deterministic equations*) are *β*_*I*_ = 0.14 [0, 0.26] in the community, *β*_*H*_ = 0.29 [0, 0.92] in the hospitals, and *β*_*F*_ = 0.40 [0, 0.99] at the funerals, where the intervals correspond to the values used to obtain the average rates of transmission obtained from the Akaike criterion. From these rates, we construct the next-generation matrix^32^,^33^ (see *Methods: Estimation of R*_0_) in order to compute the reproductive number *R*_0_, defined as the average number of people in a susceptible population one infected individual infects during his or her infectious period. This parameter is fundamental when predicting whether a disease can reach a macroscopic fraction of individuals^34^. For a critical value *R*_0_ = 1 there is a phase transition below which no epidemic takes place, and the disease is only a small outbreak, while for *R*_0_ > 1 the probability that an epidemic spreading develops is greater than zero^34^. For the values of rates of transmission given above, we find that the reproductive number of the current EVD outbreak is *R*_0_ = 2.11 [1.88, 2.71], well above the critical threshold *R*_0_ = 1, where the interval was obtained from the transmission coefficient selected from the Akaike criterion. This value of *R*_0_ is compatible with the one obtained by Rivers *et al.*^15^. We run our stochastic simulations presented in *Methods: Stochastic model* for these estimated values in order to compare the total number of cases with the data given by WHO before the interventions began in the middle of August 2014^13^. Figure 2(a) plots the number of cumulative cases as a function of time for 1000 realisations of our stochastic model and compares the results with WHO data^3^ without any shift correction. The individual realisations have the same shape as the data but due to the stochasticity at the beginning of the outbreak the exponential increase in the number of cases occurs at different moments.

Figure 2(b) plots the cumulative number of cases as a function of time with the initial conditions explained above when a temporal shift is applied to the stochastic simulations. The agreement between the simulations and the data indicates that our model can successfully represent the dynamics of the spreading of the current Ebola outbreak in Liberia.

### The geographical spread of Ebola cases across Liberia due to mobility

**Figure 3:**
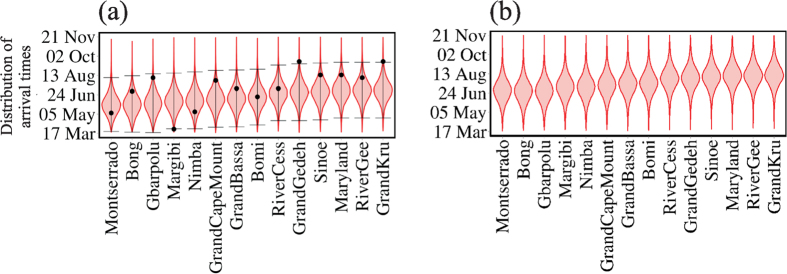
(a) Violin plots representing the distribution of arrival time *t*_*a*_ to each county considering the mobility flow of individuals among counties^21^ without any restriction on the mobility. The results are obtained from our stochastic model with the estimated transmission coefficients over 1000 realisations. Error bars indicate the 95% confidence interval. From WHO’s reports the index case (patient zero) was located at Lofa at 17 of March 2014. The circles represent the values of *t*_*a*_ reported by WHO. The very early case of Margibi is below the 5% probability, and it is explained in Ref. 35. (b) Violin plots representing the distribution of arrival time *t*_*a*_ to each county reducing the mobility among counties by 80%.

The mobility among the 15 counties allows us to compute the arrival time *t*_*a*_ in each county, assuming that the index case was in Lofa on 17 March 2014. Figure 3 shows the violin plots of the arrival times *t*_*a*_ of the disease as it spreads from Lofa County into the other 14 Liberian counties and compares our results with those supplied in the WHO reports (circles).

Comparing the results of our predictions of the arrival times of the first case as it spreads to the other counties with the WHO data (see Fig. 3a), all counties except Margibi and Grand Gedeh fall into a 95% confidence interval. This could be caused by (i) an underestimation of the number of cases in the WHO data Ref. 3 due to a lack of information^1^, or (ii) because the data recorded are actually the times of reporting and not the times of onset.

As the disease began to spread, population mobility decreased. This was in part due to imposed regulations attempting to contain the disease but also due to the population’s fear of contagion. We reflect this in our model by decreasing the mobility value. Figure 3(b) shows the arrival times produced by our model when, as a strategy for slowing the spread, the mobility is reduced by 80%. Note that this reduction delays the arrival of EVD in each county by only a few weeks. This suggests that reducing the mobility of the individuals between counties will not stop the spread but will slow it sufficiently that other strategies can be developed and applied. Reducing mobility is also insufficient when considering international transmission of the disease^19^ and more aggressive interventions are needed. We believe that an increase in both the percentage of infected individuals receiving hospitalisation in ETCs and the percentage of burials that follow procedures that do not transmit the disease are essential in containing the epidemic.

### Interventions and time to extinction

To contain the disease and reduce its transmission we reduce mobility by 80%, increase the number of burials following procedures that do not transmit the disease, and increase the rate of hospitalisation in ETCs. Because health workers in ETCs have specialised training, we assume that the probability that they will be infected is greatly reduced and that the transmission coefficient *β*_*H*_ is decreased. A sufficiently rapid response to the EVD by the ETCs requires that *β*_*H*_ be decreased exponentially to a final value of 10^−3^, and hospitalisation *θ* must be increased exponentially to reach *θ* = 1. On the other hand, when changing local burial customs we assume that *β*_*F*_ decreases linearly and approaches zero. Changing local burial customs involves recruiting and training burial teams, takes a longer period of time, and is less aggressive than other kinds of intervention. This approach allows us to estimate an upper limit for the end of the epidemic, because we did not take into account other measures applied, such as, contact tracing, which could make the estimated end to the epidemic occur earlier.

We apply these changes to simulate a two-month period, and the final result is that *R*_0_ decreases from 2.11 to *R*_0_ = 0.69, which is below the epidemic threshold. We consider two scenarios, (i) implementing the strategy beginning August 15 (the middle of the month indicated by WHO^36^ for the outbreak of EVD in West Africa) in which all symptomatic individuals are admitted to ETCs and safe burial procedures begin to apply, or (ii) implementing the same strategy, but beginning July 15 in order to study how delaying the implementation of the strategies affected containment. Our goal is to demonstrate that if the international response had been more rapid, the spreading disease would have been contained with a 50% probability by early March 2015 instead of the end of May 2015.

**Figure 4:**
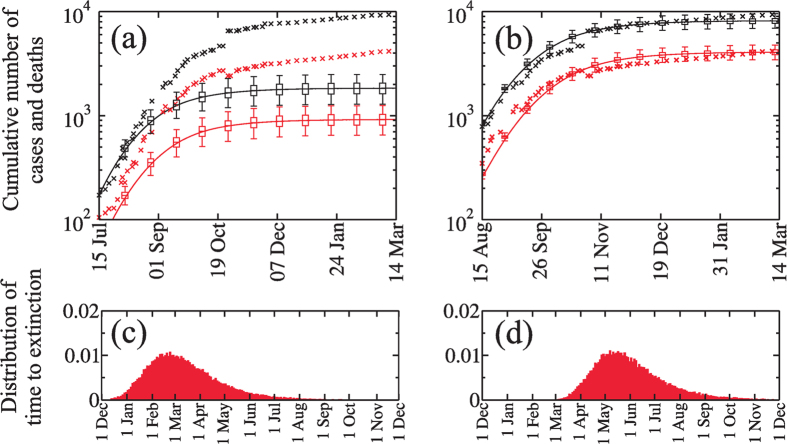
Evolution of the number of cases (black) and deaths (red) when a reduction of 80% in the mobility rates is applied. The value of *β*_*H*_ decreases exponentially to reach the value 10^−3^ and *β*_*F*_ decreases linearly to reach a 0% of their original values. Also the hospitalisation fraction increases exponentially to reach *θ* = 1. All reductions in the transmission coefficient were applied during two months, for (**a**) beginning at July 15*th* and (**b**) August 15*th*. Solid lines were obtained from the evolution equations (1, 2, 3, 4, 5, 6, 7, 8, 9, 10) and the symbols are the data. The box plots show the median, the 25th and 75th percentile and 95% confidence interval of the median, obtained from the stochastic simulations. (**c**) and (**d**) are the distribution of time to extinction of the EVD epidemic obtained from the stochastic simulations, when the strategy is applied from middle July and from middle August 2014, respectively. We show these distributions from December 1st, 2014 to December 1st, 2015.

Figure 4(a,b) show that reducing the number of cases produced in hospitals and funerals reduces the cumulative number of cases to a plateau lower than the one predicted when no strategies are applied^37^. Figure 4(a) shows that if our strategy had been applied in the middle of July the cumulative number of cases and deaths would have been approximately 80% lower than the reported number that resulted when the strategies were instituted in the middle of August. Figure 4(b) shows that when we apply the strategy of our model to the actual mid-August starting time, it predicts (between the 95% confidence interval of the median) the actual trend of cases and deaths reported in the WHO data in mid-March 2015.

Our stochastic model allows us to quantify how the two different strategy implementation times affect the extinction time of the EVD epidemic. Figure 4(c,d) show the extinction time distributions, i.e., when *E* = *I* = *H* = *F* = 0, when the strategy is implemented in July 2014 and August 2014, respectively (for the initial conditions, we use the cases provided by WHO for these dates). We find that the median of this distribution when the strategy is implemented in July is 6 March 2015 (with a 95% confidence interval from 5 January 2015 to 1 July 2015) and when it is implemented in August is 25 May 2015 (with a 95% confidence interval from 28 March 2015 to 20 September 2015). Implementation in mid-August generated 8,000 cases of the disease, but an implementation in mid-July would have reduced the time to disease extinction by three months and generated only 1,700 cases. The mid-August implementation faced a larger number of cases, the disease progression had a greater inertia against the strategy, and the cumulative number of cases required a longer time to go from an exponential regime to a subexponential regime. Thus if the health authorities and the international community had acted sooner the number of infected people would have been much lower.

## Discussion

In this manuscript we study the spreading of the Ebola virus using stochastic and deterministic compartmental models that incorporate the mobility of individuals between the counties in Liberia. We find that our model describes well the arrival of the disease into each of the counties, that reducing population mobility has little effect on geographical containment of the disease, and that reducing population mobility must be accompanied by other intervention strategies. We thus examine the effect of an intervention strategy that focuses on both an increase in safer hospitalisation and an increase in safer burial practices. Our study indicates that the intervention implemented in August 2014 reduced the total number of infected individuals significantly when compared to a scenario in which there is no strategy implementation, and it predicts that the epidemic will be extinct by mid-spring 2015. We also use our model to consider the difference in outcome had the strategy been implemented one month earlier. We find that the cumulative number of cases and deaths would have been significantly lower and that the epidemic would have ended three months earlier. This indicates that a rapid and early intervention that increases the hospitalisation and reduces the disease transmission in hospitals and at funerals is the most important response to any possible re-emerging Ebola epidemic.

Although our model simplifies the dynamics of epidemic spreading, it provides an adequate picture of the evolution in the number of cases and deaths. In future research we will incorporate more aspects of population mobility and intervention strategies carried out by health authorities. This will enable us to describe in greater detail the evolution of an epidemic and the efficacy of different strategies.

Finally, the methods used in this manuscript to study Liberia can also be applied to Guinea and Sierra Leone as soon as high quality epidemic data from those countries become available. Future work should include both countries in order to quantify the cases spreading from them into Liberia.

## Methods

### Stochastic model

We generate a stochastic compartmental model based on the Gillespie algorithm. At each iteration of the simulation we draw a random number *τ* (which represents the waiting time until the next transition) from an exponential distribution with parameter Δ given by parameter
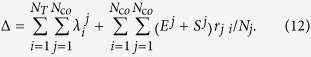


Here the first term 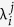 is the rate of transition between states *i* in county *j* given in Table S1, and the second term corresponds to the mobility rates given in Eq. (11) with *x* = *E* and *x* = *S*.

### Calibration with the deterministic equations

To estimate the transmission coefficients *β*_*I*_, *β*_*H*_, and *β*_*F*_ we calibrate a system of differential equations using least-squares optimisation with the data of the total cases from Liberia in the March-August period^3^, and we apply a temporal shift, which we will explain below. We compute the least-square values using a set of parameters generated using Latin hypercube sampling (LHS) in the parameter space [0, 1]^3^, which we divide into 10^6^ cubes of the same size. For each cube we choose a random point as a candidate for (*β*_*I*_, *β*_*H*_, *β*_*F*_) in order to compute the standard deviation between the data and the system of differential equations obtained from this point. At the beginning of the epidemic there are very few cases (infected individuals), thus the evolution of the disease is in a stochastic regime in which the dispersion of the number of new cases is comparable to its mean value (see Refs 38, 39, 40. When the number of infected individuals increases to a certain level, however, the epidemic evolves toward a quasi-deterministic regime and the evolution of the states of the stochastic simulation is the same as the states obtained using the solution of the evolution equations (Eqs 1, 2, 3, 4, 5, 6, 7, 8, 9, 10). Nevertheless, due to fluctuations in the initial stochastic regime, a random temporal displacement of the quasi-deterministic growth of the number of accumulated cases is generated. Thus to remove this stochastic temporal shift and to compare the three aspects—the simulations, the numerical solution, and the data—we set the initial time at *t* = 0 when the total number of cases is above a cutoff *s*_*c*_^38^,^39^,^40^. For the calibration of the transmission coefficients, we use *s*_*c*_ = 200 (which corresponds to the cumulative number of cases after 21 July, according to the WHO data^3^), and using the least square method we give the data above this cutoff 50% of the weight because we are assuming that above *s*_*c*_ the evolution of the disease spreading is quasi-deterministic. Finally, after we compute the sum of square residuals for each point in the parameter space, we apply the Akaike information criterion (AIC) and average those candidates of (*β*_*I*_, *β*_*H*_, *β*_*F*_) with a AIC difference Δ < 2^41^ to obtain a model-averaged estimate of the transmission coefficients. An alternative method for estimating the transmission coefficients using an exponential fitting is discussed in Supplementary Information*: Calibration*. We find that this fitting generates the same set of values of transmission coefficients than the method with a temporal shift *s*_*c*_. Additionally, in Supplementary Information*: Sensitivity Analysis* we discuss the sensitivity of the estimated values of the transmission coefficients when *θ* and *δ* change.

The mobility data for the Wesolowski model were provided by Flowminder^21^,^42^,^43^ and the *total cumulative* case data used to calibrate the model were those supplied in reports generated by WHO^3^. Note that in this work we do not calibrate the model to cases in each county because it was shown by Chowell *et al.*^44^ that globally the number of cases grows exponentially but locally can be better approximated by a polynomial than by an exponential growth. This cannot be addressed using our model because mathematically a differential equation with constant rates only reproduces an exponential growth.

Weitz and Dushoff^45^ recently demonstrated that calibration causes an identification problem, i.e., that many combinations of the coefficient transmission values reproduce the real evolution of the number of cases, which is compatible with our finding that the calibrated transmission coefficients are in a plane (see Supplementary Information: Calibration). This point should be addressed in future research.

### Estimation of *R*_*o*_

In order to compute the reproduction number *R*_0_, following van den Driessche *et al.*^32^ and Diekmann *et al.*^33^, we construct a next-generation matrix.

First, using the Jacobian matrix of the system of Eqs (1, 2, 3, 4, 5, 6, 7, 8, 9, 10) we construct the “transmission matrix” **F**, and the “transition matrix” **V**, obtaining
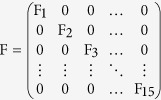
where
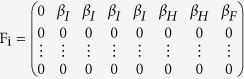
and
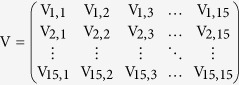
where
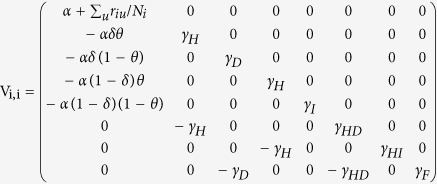

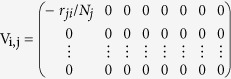
with *i* ≠ *j*.

Note that the mobility rates are only in the transition matrix. Using these matrices we construct the next generation matrix, defined as **FV**^−1^. Finally, the reproduction number is given by the spectral ratio *ρ* of the next generation matrix, *R*_0_ = *ρ*(**FV**^−1^), i.e., its highest eigenvalue. Note that when the mobility rates go to zero, 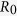 decreases, i.e., in this limit an infected individual in a given county cannot interact with people from other counties and can only transmit the disease to susceptible individuals in the same county.

## Additional Information

**How to cite this article**: Valdez, L.D. *et al.* Predicting the extinction of Ebola spreading in Liberia due to mitigation strategies. *Sci. Rep.* **5**, 12172; doi: 10.1038/srep12172 (2015).

## Supplementary Material

Supplementary Information

## Figures and Tables

**Table 1:** Transition parameters used to calculate the transition rates in our epidemic model.

**Transition Parameters**	**Value**	**References**
Mean duration of the incubation period (1/*α*)	7 days	26, 27, 28
Mean time from the onset to the hospitalisation (1/*γ*_*H*_)	5 days	29
Mean duration from onset to death (1/*γ*_*D*_)	9.6 days	29
Mean time from onset to the end for the cured (1/*γ*_*I*_)	10 days	28,30
Mean time from death to traditional burial (1/*γ*_*F*_)	2 days	14
Proportion of cases hospitalised (*θ*)	50%	15
Fatality Ratio (*δ*)	50%	15
Mean time from hospitalization to end for cured (1/*γ*_*I*_)	5 days	14
Mean time from hospitalization to dead (1/*γ*_*HD*_)	4.6 days	14

Table describing the different parameters used to calculate the transition rates among the ten different compartmental states in our model.
