# Supplementary material for: Predicting the extinction of Ebola spreading in Liberia due to mitigation strategies
Source: Sci Rep. 2015 Jul 20;5:12172. doi: 10.1038/srep12172 (PMC4507172; doi:10.1038/srep12172)
Supplement: Supplementary Information [file srep12172-s1.pdf]

Predicting the extinction of Ebola spreading in Liberia due to mitigation strategies

L. D. Valdez,[,*] H. H. Aragão Rêgo, H. E. Stanley, and L. A. Braunstein

Supplementary Information

Calibration

In the main text, using the least square method and a temporal shift for the number of cases ($s_c = 200$) we obtained the coefficient parameters β_I, β_H and β_F. We can also obtain these transmission coefficients by measuring the parameter η that best fits an exponential growth $I(t) \sim \exp(\eta t)$ in the deterministic regime. For the epidemic spreading in Liberia, we find that fitting with an exponential function on the number of cases from July 21st to August 15th, $\eta = 0.053 \pm 0.003$. To find the relation between η and the transmission parameters β_I, β_H, and β_F, we write the equation of the eigenvalue of the Jacobian J of the system of Eqs. (1)–(10) for the $N_{co} = 15$ counties

$$det\Big(J(\beta_I, \beta_H, \beta_F) - \eta\, Id\Big) = 0, \tag{S1}$$

where det is the determinant function, η is the eigenvalue obtained from the fitting, Id is the identity matrix with $10N_{co}$ rows and columns, where the factor 10 corresponds to the number of evolution equations for each county. Note that J is a function of the transmission coefficients.

Equation (S1) sets the relationship between the exponential growth rate (η) and the transmission parameters, which is not linear when the mobility is taken into account. However, we can approximate this equation by neglecting the flow of individuals. This is the case because although mobility spreads the EVD throughout the country in the stochastic stage, when the disease reaches the deterministic regime its spreading is primarily due to infected individuals within each county and imported cases are no longer a relevant factor. With this approximation, and using $\eta = 0.054$, we obtain an equation of a plane (see Fig. S1) that coincides with the triads of transmission coefficients that were obtained by using the least square fitting with a shift s_c. Thus this method

*Electronic address: ldvaldes@mdp.edu.ar

and the exponential fitting generate the same set of transmission parameters that reproduces the epidemic growth.

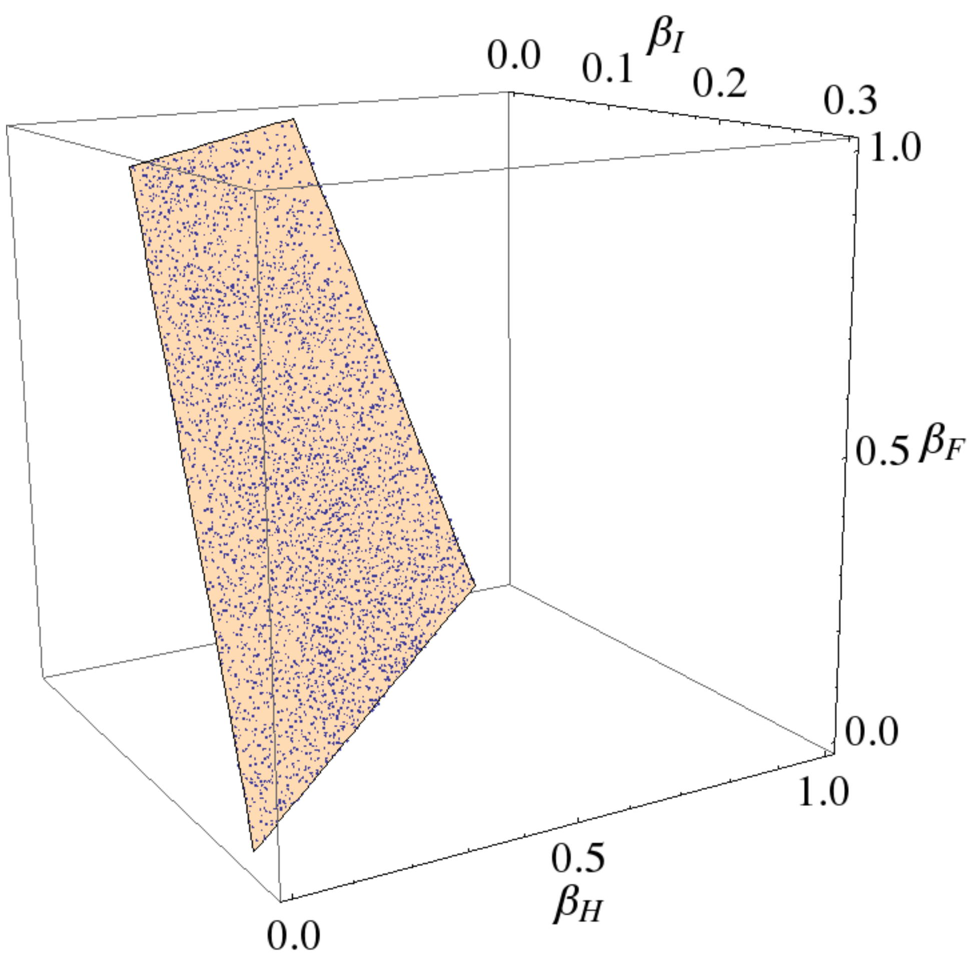

FIG. S1: Values of $(\beta_I, \beta_H, \beta_F)$ that fits the data obtained from i) the shift and least square method (points) and ii) fitting the exponential growth of number of cases (plane).

Note that in the main text we use $s_c = 200$ to calibrate the data. On the other hand, if we use $s_c = 100$ (near the 2 July date) we obtain $\beta_I = 0.09$ $(0, 0.23)$, $\beta_H = 0.31$ $(0, 0.78)$, and $\beta_F = 0.46$ $(0, 0.99)$, and if we use $s_c = 300$ (near the 28 July date) we obtain $\beta_I = 0.11$ $(0, 0.27)$, $\beta_H = 0.38$ $(0, 0.94)$ and $\beta_F = 0.46$ $(0, 0.99)$.

Table of the transitions

Transition	Transition rate (λ_i)
$(S, E) \to (S-1, E+1)$	$\frac{1}{N}(\beta_F\ S\ I + \beta_H\ S\ H + \beta_F\ S\ F)$
$(E, I_{DH}) \to (E-1, I_{DH}+1)$	$\alpha\ \theta\ \delta E$
$(E, I_{DNH}) \to (E-1, I_{DNH}+1)$	$\alpha\ (1-\theta)\ \delta E$
$(E, I_{RH}) \to (E-1, I_{RH}+1)$	$\alpha\ \theta\ (1-\delta)\ E$
$(E, I_{RNH}) \to (E-1, I_{RNH}+1)$	$\alpha\ (1-\theta)\ (1-\delta)\ E$
$(I_{DH}, H_D) \to (I_{DH}-1, H_d+1)$	$\gamma_H\ I_{DH}$
$(I_{DNH}, F) \to (I_{DNH}-1, F+1)$	$\gamma_D\ I_{DNH}$
$(I_{RH}, H_R) \to (I_{RH}-1, H_R+1)$	$\gamma_H\ I_{RH}$
$(I_{RNH}, R) \to (I_{RNH}-1, R+1)$	$\gamma_I\ I_{RNH}$
$(H_D, F) \to (H_D-1, F+1)$	$\gamma_{HD}\ H_D$
$(H_R, R) \to (H_R-1, R+1)$	$\gamma_{HI}\ H_R$
$(F, R) \to (F-1, R+1)$	$\gamma_F\ F$

TABLE S1: **Table of the transition with their respective transition rates for our model.** Table representing the transition rates between different compartmental states in our model. The capital letters represents number of: susceptible individuals (S), number of exposed individuals (E), individuals infected who will be hospitalised and die (I_{DH}), individuals infected who won't be non hospitalised and will die (I_{DNH}), individuals infected who will be hospitalised and recovered (I_{RH}), individuals infected who won't be hospitalised and will recover (I_{RNH}), individuals hospitalised who will die (H_D), individuals hospitalised who will recover (H_R). Here R is the number of individuals cured or dead and F is the number of individuals in the funerals who will have unsafe burials and can infect. Here β_I, β_H and β_F are the transmission coefficients in the community in the hospital and in the funerals respectively, δ is the fatality ratio and θ the fraction of the hospitalised ones. The inverse of the mean time period of the incubation is $1/\alpha$. The mean time period from symptoms to hospitalisation is $1/\gamma_H$, from symptoms for non hospitalised individuals to dead is $1/\gamma_D$, from symptoms for hospitalised individuals to dead is $1/\gamma_{HD}$, from symptoms for hospitalised individuals to recovery is $1/\gamma_{HI}$ and from dead to recover is $1/\gamma_F$. The flow of mobility for individuals in county $i \to j$ is explained in Eq. (11).

Sensitivity analysis

Because the epidemic evolution in this model is affected by many parameters, small changes in their values could significantly impact the model's output. Here we analyse how changing the hospitalisation ratio θ and the death ratio δ affects the estimation of β_I, β_H, β_F, and R_0, and also affects the evolution of the cumulative cases when the strategy is implemented in August as described in the main text.

For θ and $\delta = 0.40,\ 0.50,\ 0.60$ we show the values of β_I, β_H, β_F, and R_0, using the least square method and the Akaike average, as explained in the Methods section.

θ	β_I	β_H	β_F	R_0
0.40	0.13	0.37	0.40	2.24 (1.99,2.28)
0.50	0.14	0.29	0.40	2.11 (1.88,2.71)
0.60	0.14	0.25	0.39	2.05 (1.92,2.28)

TABLE S2: Values of the transmission coefficients and R_0 for $\theta = 0.40,\ 0.50,\ 0.60$. Here $\delta = 0.5$

δ	β_I	β_H	β_F	R_0
0.40	0.14	0.30	0.42	2.18 (1.97,2.16)
0.50	0.14	0.29	0.40	2.11 (1.88,2.71)
0.60	0.13	0.29	0.38	2.05 (1.96,2.63)

TABLE S3: Values of the transmission coefficients and R_0 for $\delta = 0.40,\ 0.50,\ 0.60$. Here $\theta = 0.5$

Table S2 shows that as θ increases 50% from $\theta = 0.40$ to $\theta = 0.60$, β_H decreases 30%. In contrast, β_I and β_F remain almost constant and the reproductive number does not change significantly. On the other hand, Table S3 shows that when δ increases from $\delta = 0.40$ to $\delta = 0.60$, the transmission coefficients and R_0 change less than 10%, however it remains inside the interval. Thus this model is more sensitive to changes in θ than in δ. Figure S2 plots the number of cumulative cases as a function of time obtained from the stochastic model, compares the results with WHO data, and shows good agreement between the simulations and the real data.

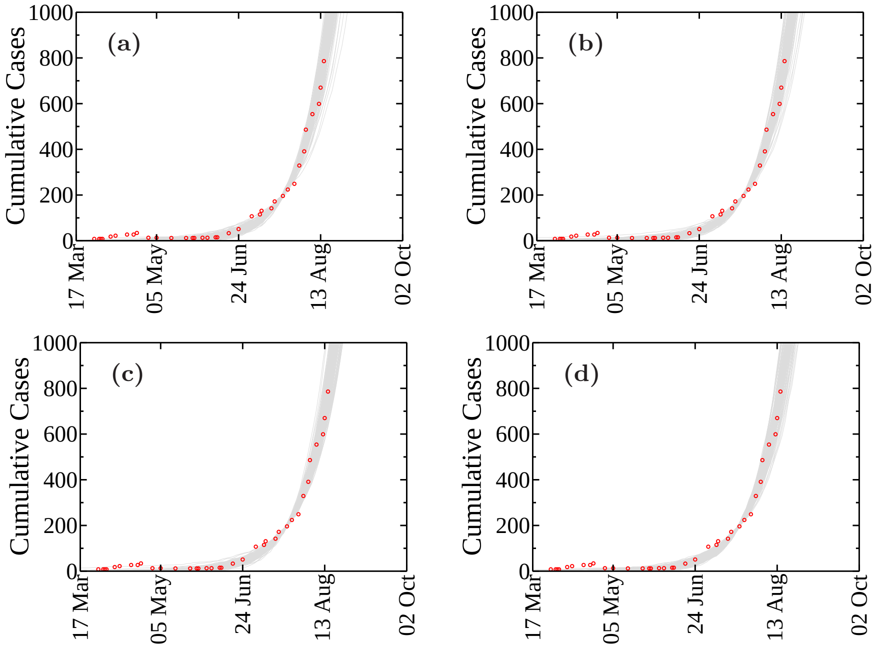

FIG. S2: Evolution of the cumulative number of cases in Liberia with 100 realisations (gray lines) and the data (symbols) with a temporal shift using $s_c = 200$. Figures (a) and (b) correspond to $\theta = 0.40$ and $\theta = 0.60$ respectively, with $\delta = 0.5$. The transmission coefficients used, were obtained from table S2. Figures (c) and (d) correspond to $\delta = 0.40$ and $\delta = 0.60$, respectively, with $\theta = 0.50$. The transmission coefficients used, were obtained from table S3.

To evaluate how changing the parameter values alters the effectiveness of the intervention strategy as explained in the main text, Fig. S3 plots the cumulative number of infected individuals when the strategy is implemented in mid-August for different values of θ and δ.

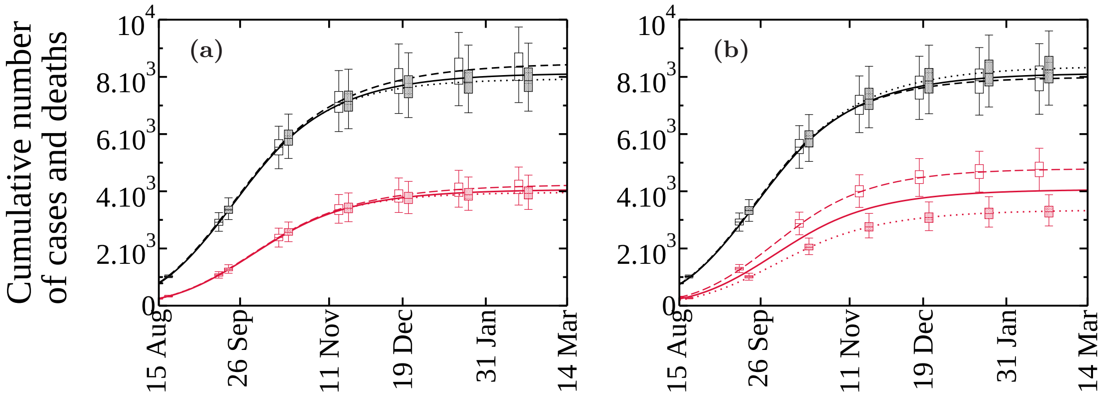

FIG. S3: Evolution of the cumulative number of cases (black) and deaths (red) when it is applied the strategy from August, as it was explained in the main text, for different values of θ and δ. In the figure (a), $\delta = 0.50$ and $\theta = 0.50$ (solid line), $\theta = 0.40$ (dotted line and filled box plots) and $\theta = 0.60$ (dashed line and open box plots). In figure (b) $\theta = 0.50$ and $\delta = 0.50$ (solid line), $\delta = 0.40$ (dotted line and filled box plots) and $\theta = 0.60$ (dashed line and open box plots). All the curves were obtained by integrating the evolution equations (1)-(10), and the box plots were obtained from the stochastic simulations.

Figure S3 shows that although the evolution of the number of *cases* is not sensitive to variations in θ and δ, the evolution of number of *deaths* is sensitive to variations in δ (see Fig.S3b). This is the case because the final number of deaths is proportional to δ, and in our strategy β_F changes but δ remains constant.
